# Understanding Determinants of Electronic Cigarette and Heated Tobacco Product Use among Young Adults in Lebanon: Prevention and Policy Implications

**DOI:** 10.3390/ijerph20054273

**Published:** 2023-02-28

**Authors:** Sanaa Mugharbil, Malak Tleis, Maya Romani, Ramzi G. Salloum, Rima Nakkash

**Affiliations:** 1 Department of Health Promotion and Community Health, Faculty of Health Sciences, American University of Beirut, Beirut 11-0236, Lebanon; 2 Department of Family Medicine, Faculty of Medicine, American University of Beirut Medical Center, Beirut 11-0236, Lebanon; 3 Department of Health Outcomes and Biomedical Informatics, College of Medicine, University of Florida, Gainesville, FL 32611, USA; 4 Global and Community Health Department, College of Public Health, George Mason University, Fairfax, VA 22030, USA

**Keywords:** e-cigarettes, HTP, determinants, deterrents, qualitative

## Abstract

In line with the global trends, electronic cigarettes (e-cigarettes) and heated tobacco products (HTPs) have found their way to the Lebanese market. The present study aims to explore the determinants of e-cigarette and HTP use among young adults in Lebanon. Convenience and snowball sampling were used to recruit participants aged 18–30 residing in Lebanon, who were familiar with e-cigarettes products. Twenty-one consenting participants were interviewed via Zoom and the verbatim transcriptions were analyzed thematically. The outcome expectancy theory was used to categorize the results into determinants and deterrents of use. HTPs were viewed by participants as another mode of smoking. The results showed that most participants perceived e-cigarettes and HTPs to be healthier alternatives to cigarettes/waterpipes and to be used as smoking cessation tools. Both e-cigarettes and HTPs were found to be easily accessible in Lebanon; although, in the recent economic crisis, e-cigarettes have become unaffordable. More research is needed to investigate the motivations and behaviors of e-cigarette and HTP users if effective policies and regulations are to be developed and enforced. Furthermore, greater public health efforts need to be made to increase awareness of the harmful impacts of e-cigarettes and HTPs and to implement evidence-based cessation programs tailored to those modes of smoking.

## 1. Introduction

Electronic nicotine delivery systems (ENDS), referred to here as e-cigarettes, are devices that deliver nicotine to the consumer and allow them to mimic the sensory effects of smoking without burning tobacco and inhaling the smoke from the combustion as one does with tobacco cigarettes [1]. Similarly, heated tobacco products (HTPs) are tobacco products that produce aerosols containing nicotine and other chemicals. Rather than heating e-liquids, as is done with e-cigarette products, HTPs heat up tobacco. To date, research on the use of ENDS among youths has mostly been conducted in high-income countries. A literature review of 27 studies found prevalence data only in 13 countries, with 10 of the 27 studies being from the US [2].

E-cigarettes have been marketed as less harmful than tobacco cigarettes because of the claim that they contain lower levels of toxicants and that they are smoking cessation aides [3,4]. Youths and young adults are among the primary target demographics for ENDS, with celebrity endorsements and the promotion of flavored products being used to appeal to the younger generation [2]. The impact of this is perhaps evident when considering that e-cigarette users are more likely to be youths [5]. A case in point is a recent lawsuit, initiated by nearly 30 US states, against JUUL Labs, a top US e-cigarette company. JUUL Labs is set to pay $438.5 million to settle a lawsuit investigating the company’s advertising, which has been accused of targeting underage buyers [6].

Although to date no prevalence data exist for e-cigarette use in Lebanon, the prevalence of cigarette smoking in Lebanon is documented as being among the highest in the world, with 35.1% of males and females smoking cigarettes [7]. It also has one of the highest prevalences (39.5%) of waterpipe smoking [7]. Although a comprehensive tobacco control policy law came into effect in Lebanon in 2011, smoking prevalence remains largely unimpacted due to the weak enforcement of the law [8]. E-cigarettes remain unregulated and uncontrolled in any way, once introduced to the market. Exacerbating the situation is the severe economic crisis that Lebanon is witnessing which has deprioritized tobacco control [9]. The present study aims to explore the determinants and drivers of e-cigarette use among Lebanese young adults to inform its future potential prevention and policy efforts.

## 2. Materials and Methods

### 2.1. Participant Recruitment

This study is a qualitative study which followed a qualitative research methodology. It used convenience sampling as the primary method of recruitment. Following approval from the American University of Beirut’s Institutional Review Board (approval number: SBS-2020-0386), flyers advertising the study objective, recruitment details, information on incentives, and contact information were posted on Facebook and Instagram, between May 2021 and June 2021. Snowball sampling was used as a secondary means of recruitment where people were asked to forward the study invite to others they thought would be interested and willing to participate. An incentive, in the form of phone credit valued at 15 USD, was given following participation.

After posting the flyer, 31 respondents completed the online survey, all of whom were contacted for the interview. Interested participants were directed to a link for eligibility screening. The eligibility criteria included: (1) being of ages between 18–30 years old, (2) residing in Lebanon, and (3) being familiar with e-cigarettes regardless of their use of these products. Among the total respondents, 1 was under 18 years of age, 1 refused to do the interview, 2 skipped the interview, and 6 did not respond to messages or calls, resulting in a total of 21 eligible participants—12 females and 9 males. The eligible participants were first contacted by phone to review and explain the objective and details of the study and the elements of the informed consent forms. The final date and time were then set for the interview. Electronic consent forms were sent to all participants to read and approve. In addition to outlining issues of confidentiality, anonymity, and voluntariness, the participants were asked to consent to participation, and then provide consent for the recording of the interview and for the use of individual quotes (without personal identifiers) in the reporting of the findings.

### 2.2. Data Collection

The eligible participants were directed to complete a short online survey which captured their socio-demographic information and their e-cigarette use. Semi-structured interviews were then conducted over Zoom, following an interview guide of open-ended questions in which participants were asked about their perceptions on e-cigarettes, their patterns of use, comparisons to other tobacco and nicotine products, the marketing of these products, and their future intentions concerning use. The interviews took 20 min on average. The data analysis was iterative; therefore, once data saturation was reached at 21 interviews, no further interviews were conducted.

### 2.3. Data Analysis

The interviews were recorded then transcribed verbatim by a hired research consultant. A thematic analysis was then conducted, and a codebook was developed. Both deductive and inductive coding were used. Two interviews were coded by SM and MT independently. Comparisons of the independent coding showed similar codes by both coders. Following multiple rounds of discussions and revisions by SM, RN, and MT, the research team reached an agreement on all the final themes and a codebook was developed.

## 3. Results

**Figure 1:**
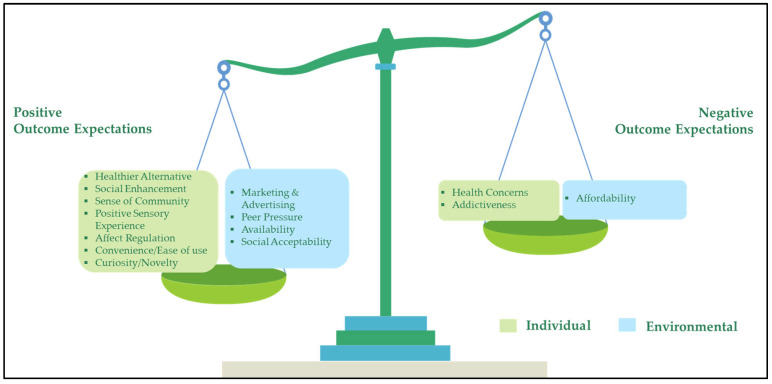
Participants’ positive versus negative outcome expectations at the individual and environmental level.

The results were themed according to outcome expectancy, which is a factor that has been found to play a significant role in substance abuse behavior [10]. It refers to the expected outcomes resulting from a certain behavior [10], where positive or favorable outcomes increase the likelihood of that behavior, and negative or unfavorable outcomes decrease the likelihood of it [11]. Based on this theory, the results were divided into determinants, i.e., factors which increased the likelihood of vaping due to positive expectations, and deterrents, i.e., factors which decreased the likelihood of vaping due to negative expectations. An adaptation of the socio-ecological model was used to further categorize these factors into individual and environmental levels (Figure 1).

The demographic characteristics of the participants are summarized in Table 1.

A cross-cutting finding that is important to note is that, although the aim of the study was to focus on the determinants and drivers of e-cigarette use, several participants also mentioned HTPs in their discussion, namely IQOS products, which had been widely marketed around the time of the data collection. We distinguish the findings pertaining to e-cigarette versus HTP use when possible and indicate when participants were referring to both by providing relevant quotations.

The emerging themes in which both e-cigarettes and IQOS were mentioned included the substitution of cigarette smoking, positive sensory experience, convenience/ease of use, curiosity and novelty, marketing and advertisement, availability, health concerns, and addictiveness. For the remaining themes, i.e., social enhancement, affect regulation, peer pressure, social acceptability, and affordability, only e-cigarettes were mentioned.

The brand IQOS was the only HTP product referred to by the participants. Consequently, the term IQOS is used throughout.

### 3.1. Determinants Increased Likelihood of Vaping Due to Positive Expectations

#### 3.1.1. Individual Factors

##### Healthier Alternative

The most common determinant for initiating both e-cigarette and/or IQOS use was the perception that they are healthier alternatives to cigarettes and waterpipes.

*… we don’t have the combustion [in reference to vapes], we don’t have the carbons that come out of the combustion.—male, 23, e-cigarette user*

*… [with] IQOS, there isn’t this coughing or those irritating things [like with tobacco cigarettes]—female, 21, IQOS product user*

Although some participants acknowledged the negative health impacts that vapes/IQOS products have, they were still seen as healthier than tobacco cigarettes.

Smoking everything is bad. But maybe there are things more harmful than others.—female, 25, e-cigarette user

In fact, many reported using e-cigarettes/IQOS products to quit or reduce smoking cigarettes and/or waterpipes. Many others reported that their peers, family members, and even their physicians, encouraged them to switch to e-cigarettes/IQOS products for smoking cessation.

*the doctors say “… Stop smoking [cigarettes] and turn to this [vaping]. Stick to this, it’s less harmful”.—male, 23, e-cigarette user*

##### Social Enhancement

Wanting to fit in and being accepted within a certain social group were repeatedly reported as motivators for initiating e-cigarette use.

*They think it’s [vaping] cool … they see … people [and think] like oh if I want to be cool … like oh I’m bad I break rules …—female, 20, e-cigarette user*

This notion was echoed by other participants who mentioned that e-cigarettes gave one a sense of confidence and portrayed a certain desirable image.

##### Sense of Community

Participants were also attracted to e-cigarettes for the sense of community or belonging they bring. Online forums, Facebook pages, WhatsApp groups, and face-to-face events were all mentioned as mediums in which e-cigarette users discuss their products, recommend flavors, and support each other.

*We have a huge community, especially in Lebanon. We used to be like from 5 to 6 vapers, we are now around 3–4000 vapers … we all get in contact mainly weekly or monthly through selling devices that we introduce to each other … We all bonded through this electronic device.—male, 23, vape user*

##### Positive Sensory Experience

A recurring determinant of e-cigarette use was the positive sensory experience it brings. The vast variety of flavors, the “delicious” taste, and the pleasant smell were all perceived to have a major impact on e-cigarettes’ attractiveness.

*What’s appealing about them is that there are thousands of flavors.—male, 19, e-cigarette user*

Smell was particularly important among women as it was seen as more acceptable for women to smoke e-cigarettes rather than smoke cigarettes, which emanate an unpleasant smell.

*… girls they say it’s not nice if her breath smells, it’s not nice if her clothes smell … her nails and fingers get yellow [from cigarette smoking] …—female, 23, cigarette user*

In contrast, IQOS products were frequently described as having an unpleasant smell that deterred the participants from using them.

*… very, very bad. For me, it is worse that a normal cigarette.—male, 27, e-cigarette user*

E-cigarette use was also seen as entertaining, especially in social settings, and they were seen as more of a social rather than solitary activity.

*Another type of person who goes to vape, is the type that wants … just to entertain himself for fun.—male, 25, e-cigarette user*

##### Affect Regulation

Most respondents stated that e-cigarettes were used to relieve stress, induce calmness, and/or alleviate boredom. This was reported to have increased during the COVID pandemic. Attempts to quit e-cigarette use were hindered by stress from the pandemic, work, school, and the daily stressors of living especially at the time of a major economic crisis in Lebanon.

*… the problem is that even though there was awareness [about the harmfulness of vaping], people see vaping … as a way to relieve pressure and anxiety.—male, 27, e-cigarette user*

##### Convenience/Ease of Use

Both e-cigarettes, which were frequently referred to as “mini waterpipes”, and IQOS products were seen as more convenient to use than cigarettes or waterpipes.

*… [vaping is] like a shortcut, it’s just like something which you can just put it in your pocket and just get it out and smoke it whenever you want, wherever you want.—female, 20, e-cigarette user*

The lack of confinement to where and when a person can use vapes/IQOS products was appealing particularly in the context of weakly imposed indoor smoking bans such as those in Lebanon, which in principle include e-cigarette use.

*Nowadays, we’re not tied to a certain setting … Currently we’re using these devices, small ones, pocket devices, we can use them anywhere, even in some airports … They’re usable inside of restaurants, inside of shopping malls. So, nothing can stop us or make us go outside to the outdoors to have our puffs.—male, 23, e-cigarette user*

##### Curiosity or Novelty

The novelty of e-cigarettes and IQOS products were mentioned as motivators for experimentation.

*… That’s why young people are going to these products [vapes], because … they hear that this is yummy and … delicious … So, they have the curiosity to try.—male, 28, e-cigarette user*

#### 3.1.2. Environmental Factors

##### Marketing/Advertisement

The main messages of advertisements for e-cigarettes and IQOS products seem to portray those products as healthier alternatives to cigarettes.

*… media coverage of vaping has brainwashed people into thinking that vaping is not harmful at all and that it doesn’t cause lung cancer …—male, 19, non-user of nicotine and tobacco products*

Social media trends in which influencers would do tricks with vapor/smoke made them be seen as something fun and attractive. The younger population, generally being more impressionable, would see these on social media and emulate this behavior.

*… they [vapes] are really trendy, especially on social media … Most of the guys that started, started for the trend… that’s mainly how teenagers went into vaping because of the many good tricks …many find these tricks interesting to be watching, to be participating in …—male, 23, e-cigarette user*

*There are a few vape dealers that are known. Like we search for them on Facebook or Instagram. You find them quickly.—female, 25, e-cigarette user*

##### Peer Pressure

Participants reported sometimes feeling pressured, whether advertently or inadvertently, by their friends to start using e-cigarettes.

*… if you want to talk more about preteens and early teen years … so they could be more peer pressured to go into “social norms” …—male, 19, non-user of nicotine and tobacco products*

##### Availability

All the participants reported smoking their first e-cigarette or IQOS products due to someone in their immediate social circle. Moreover, with online delivery, e-cigarettes and IQOS products were easily accessible. Although e-cigarettes were technically illegal in Lebanon and mostly smuggled, this did not impact their availability; it merely made stores more cautious about selling. Most stores sold e-cigarettes in addition to other products (e.g., electronics, clothes, etc.) and only took them out upon the customer’s request.

*… go into a phone shop, you can find the vaping devices inside, hidden under tables …—male, 23, e-cigarette user*

IQOS products on the other hand, were sold openly in major supermarkets and some minimarkets.

*[IQOS] is very much available. There are companies or stores that send you SMS: “IQOS plus HEETS for I don’t know how many dollars” … There are stores you can find it, usually online, and [the supermarket] … it is very available.—female, 26, IQOS product user*

Several participants perceived e-cigarettes to be a dying trend, especially with the introduction of IQOS products, which were perceived to be ‘trendy’ and less high maintenance than e-cigarettes.

*… But I think the vape had a certain period, maybe in 2019 … everyone was smoking vapes. But because the vape needs high maintenance…They have issues that you have to like take care of it and go fix it and do the maintenance and get a new coil this kind of stuff.—female, 25, e-cigarette user*

*… according to what I’m seeing … I feel it’s [vaping] decreased. We’re not seeing it’s widespread like before … maybe because of the economic crisis …—male, 19, non-user of nicotine and tobacco products*

##### Social Acceptability

In contrast to social enhancement, which referred to personal acceptance on an individual level, social acceptability was referred to in the context of how e-cigarettes are viewed on a general, societal level. Generally, e-cigarettes were seen to be more socially acceptable than cigarettes and waterpipes. Compared to cigarettes, using e-cigarettes indoors was also seen as more acceptable since the smell and vapor from e-cigarettes were deemed more tolerable.

*What’s nice about [vaping], it doesn’t bother anyone next to you. No one even notices that you’re vaping … when I was in class I used to vape. … An even in the winter, inside somewhere closed, no one notices, or no one gets bothered.—male, 25, e-cigarette user*

Parents were also seen to be more accepting of their children using e-cigarettes as they were perceived to be healthier than tobacco cigarettes.

Although, in general, e-cigarettes were seen as more socially acceptable, there were a handful of participants who mentioned that, in their experience, people they had encountered were opposed to e-cigarettes. This was mainly attributed to the fact that e-cigarettes work on batteries and/or electricity, which were seen as more dangerous since they are artificial as opposed to tobacco, which is “natural”.

*… [society is] scared of technology … I feel they prefer cigarettes … Not a lot of people accept the vape. Like “What is this? There’s a battery. There’s something wrong. Electricity.” So, yeah, they prefer cigarettes—this is something natural, you grow it.—male, 25, e-cigarette user*

### 3.2. Deterrents: Decreased Likelihood of Vaping Due to Negative Expectations

#### 3.2.1. Individual Level Factors

##### Health Concerns

Although many perceived e-cigarettes/IQOS products to be healthier alternatives, a smaller number acknowledged their negative health impacts.

*All [vapes, IQOS, cigarettes] have harmful diseases. Especially with vaping, it’s directly on the mouth … I heard it causes cancer …—male, 19, non-user of nicotine and tobacco products*

*… after a while, I started feeling its [vaping’s] outcomes … it [vaping] fills up inside like a liquid, in our lungs … I was very athletic; I mean, I go and run, play football and stuff like that. So … I started getting tired due to it.—male, 22, e-cigarette user*

Health concerns, although deemed to be less severe than those associated with cigarettes/waterpipe, revolved mainly around the breathing difficulties experienced by e-cigarette/IQOS product smokers. These health concerns were more prominent during the COVID-19 pandemic. The participants who used e-cigarettes and/or smoked IQOS products reported that they reduced or temporarily stopped smoking the products while infected with COVID-19.

*I had a very mild cough at that time of the corona. It was not … a severe cough but when I use it [the vape], I feel that my body is not accepting it.—male, 27, e-cigarette user*

Although health concerns were mentioned, many viewed the scientific evidence related to the health implications of e-cigarettes/IQOS products to be inconclusive.

*… cigarettes we know what they do. There is enough research to let us know what’s happening when you smoke … Vaping … there is research but not much … So, it is sort of debatable. –female, 26, IQOS product user*

Finally, e-cigarettes were seen as more susceptible to manipulation by the retailers to make them more harmful.

*…the issue is, in Lebanon, there’s a lot [e-cigarettes] that aren’t original … anyone was putting anything [in vapes] and selling it.—female, 21, IQOS product user*

##### Addictiveness

Many participants regarded e-cigarettes/IQOS products as just as addictive or slightly less addictive than cigarettes.

*… [IQOS] is addictive. I smoke [IQOS] normally, like my old habits with normal cigarette … I used to smoke 2 packs [per day] of cigarettes … Now I smoke 2 packs of IQOS. It’s the same.—female, 26, IQOS user*

However, since with e-cigarettes/IQOS products a person can control the level of nicotine they consume, these products were seen as a way to reduce people’s nicotine dependency by gradually lowering nicotine levels. This contrasts with the views of participants who believed that even with this control over nicotine levels, one would still be ingesting the same amount of nicotine, perhaps even more, than they would by smoking tobacco cigarettes because of the longer smoking time.

*… [my brother] got it [vape] so that he can reduce traditional smoking … But no. He went back to the traditional cigarettes. He didn’t decrease.—male, 19, non-user of nicotine and tobacco products*

Another point raised was that while cigarettes had a finite end after a certain number of puffs, e-cigarettes/IQOS products do not have a defined endpoint. A person can smoke as much as they want and whenever they want. Hence, attempts to smoke less, especially in terms of reducing nicotine content, were seen as futile. Some participants mentioned that although a person can put less nicotine in e-cigarettes/IQOS products, they tend to smoke more of it, so in the end they would be consuming the same amount of nicotine as they would have by smoking 20 cigarettes, for example.

*… the problem with vaping is that it does not have a session like the waterpipe … vaping is portable, available in hand so you can say that it is possible if someone wants to use it 24/7 then he can …—male, 27, e-cigarette user*

*With the vape … you don’t have a time limit like how you would have with the cigarette … with … [vaping] … you can keep on smoking … as much as possible … So, you get more nicotine than you want, and it feels bad.—female, 26, e-cigarette user*

Besides the physiological addictiveness of e-cigarettes/IQOS products, participants also mentioned the addictiveness to the behavior or action of smoking, i.e., the habit of holding something in your hands and smoking it.

*I can tell you that it is by habit and having the device in front of you more than it being a need. I mean I don’t feel the need for nicotine like with a cigarette, but I see the device in front of me, I hold it and start vaping.—male, 27, e-cigarette user*

#### 3.2.2. Environmental Factors

##### Affordability

Although e-cigarettes were seen to be easily accessible and available, following the Lebanese economic crisis and the devaluation of the currency, they were seen as not affordable. In some cases, participants went back to smoking cigarettes due to the increased prices of e-cigarette products.

*… with the dollar crisis and things like that, people aren’t using vapes a lot. They are using local cigarettes more.—male, 19, non-user of nicotine and tobacco products*

*The vape requires this machine with its charger and with its refill. So, there are costs for you to acquire it … not everyone can afford to have access to this, to this product.—female, 29, non-user of nicotine and tobacco products*

Moreover, e-cigarettes were said to require a lot of maintenance, and replacement parts are either not available in Lebanon or are too expensive.

## 4. Discussion

Comparable to findings in the literature, the results showed highly positive perceptions toward e-cigarette use with most participants reporting favorable outcome expectancies which encouraged initiation and continued use. A recurrent finding was that e-cigarettes are healthier alternatives to cigarettes and waterpipes and thus can be used as smoking cessation tools; the same was mentioned for IQOS products, which were copiously referred to by the participants. The positive sensory experience, such as that provided by the variety of flavors, the pleasant smell, and taste, was one of the most appealing aspects of e-cigarettes and attracted cigarette users as well as non-cigarette users. The ease with which e-cigarettes could be found further facilitated their use.

In concurrence with a study conducted by Jiang et al. (2019) on the perceptions of e-cigarettes among young adults in Hong Kong [12], our study found that most of the participants, particularly nicotine and tobacco product users, believed that e-cigarettes are less harmful than tobacco cigarettes. As confirmed by Glantz’s (2018) study [3], there was a belief that less exposure to chemicals means less harm. This belief was further supported by marketing campaigns in which e-cigarettes were advertised as healthier and cleaner alternatives to cigarettes [13]. Advertising and marketing campaigns played a vital role in influencing the participants’ perceptions, as others have reported as well [13]. Perceiving those products as healthier drove many smokers to use e-cigarettes and IQOS products for smoking cessation. A study conducted by Bold et al. (2016) on reasons for trying and continuing e-cigarette use found that using e-cigarettes to quit smoking was a significant predictor of continued e-cigarette use [14].

Using e-cigarettes as smoking cessation aids was found to be ineffective by some participants. Most participants stated that they or someone they knew continued to smoke cigarettes even after “switching” to e-cigarettes/IQOS products. The ability of e-cigarettes to aid in smoking cessation is a contested topic as there is little to no evidence of their effectiveness [15]. A recent systematic review found that, although ENDS products increased smoking cessation in clinical trials, this was not the case in observational studies [16].

Some participants however acknowledged the negative health impacts of e-cigarettes. These health concerns were heightened for the participants who were infected with COVID-19; the participants either stopped or decreased e-cigarette use during infection. A study on smoking patterns during COVID-19 also found health concerns to be an influential determinant associated with reduced smoking during the pandemic [17]. The perception that there is little research on e-cigarettes’ health implications also contributed to participants casting doubt on whether they were in fact unhealthy or not. The health implications of cigarettes/waterpipe smoking were seen as more conclusive than those of e-cigarette smoking. This finding was similar to that of Tompkins et al.’s (2020) study [18], in which, although participants acknowledged the harmful effects of IQOS products, due to their perceptions of there being a lack of robust and thorough research on such products, they remained optimistic about the harmfulness of HTP in comparison to that of tobacco cigarettes.

With regard to addictiveness of e-cigarettes, in contrast to similar studies [12,19], our study found that most participants believed that e-cigarettes, as well as IQOS products, are just as addictive as cigarettes. In Wilson et al.’s (2019) study, 25% of participants perceived electronic cigarettes to be less addictive than cigarettes [20]. The participants in our study also mentioned physiological addiction while highlighting the behavioral component of addiction. Tompkins et al. (2020), in their study investigating the factors that determine IQOS product use, found that participants continued to smoke IQOS products as smoking these mirrored their rituals of smoking tobacco cigarettes [18].

The sensory experience of vaping was another factor which was found to play a prominent role in motivating the use of vaping products. As confirmed by similar studies, a major determinant for using e-cigarettes was their provision of pleasant sensory experiences [21]. Flavor was by far the most mentioned attractive aspect of e-cigarettes. Flavor has also been cited as the most appealing aspect of waterpipe use among youths [22] as well as flavored or menthol cigarettes sold in the US [23].

A notable finding was that all the participants mentioned that they were first introduced to e-cigarettes by someone within their immediate social circle. This is not unusual as the most typical social sources of e-cigarettes are members of a person’s peer group [24]. Peer influence has often been cited as the most common reason for e-cigarette experimentation among youths [15]. The participants also mentioned peer influence in the form of social enhancement, in terms of wanting to fit in and to be seen as “cool” among their friends—reasons which the literature frequently cites as primary drivers of e-cigarette experimentation [14].

Some participants merely tried e-cigarettes out of curiosity and interest in the novelty of the devices after seeing friends/family members using them. Experimentation with novel devices was found to be a significant factor in several studies among high school and college students [19,21]. A lot of this curiosity also stemmed from seeing these products on various social media platforms. Social media was frequently mentioned when discussing e-cigarette smoking initiation as it related to the marketing and promotion of these products. Exposure to marketing and advertising through social media trends was seen to play an influential role in e-cigarette use, particularly among youths [25]. As with similar studies, social media influencers were seen to have a major impact on exposing youths to these products and enticing them to start using them [26]. 

Studies on the social acceptability of e-cigarettes have had mixed results [12]. In our study, and as found by Lee et al. (2017) [21], because e-cigarettes were seen as healthier, they were also seen as socially acceptable, especially in comparison to cigarettes. In fact, cigarette smokers were often encouraged by their family, friends, or sometimes their physicians to switch to e-cigarettes. Parents who were more accepting of their children smoking e-cigarettes were also mentioned in Bigwanto et al.’s (2019) study [19]. Furthermore, the pleasant flavor and smell of e-cigarettes contributed to them being more socially acceptable. Although they are generally perceived as more socially acceptable, there were some participants who experienced negative backlash regarding their use of e-cigarettes. This was mainly due to the presence of a battery or electricity in these products. They were perceived as more dangerous than “natural” tobacco. To the best of our knowledge, this result was not reflected in other similar studies.

Contrary to Bold et al. (2016) who reported the low cost of e-cigarettes as a predictor of frequent e-cigarette use [14], e-cigarettes were seen as unaffordable and a luxury in the context of our study, and this deterred their use. Despite their unaffordability, however, e-cigarettes were still perceived as easily accessible, and available. For example, e-cigarettes were sold on different social media platforms and delivered with ease [27].

E-cigarettes and similar products have been frequently found to be used for emotional regulation [10]. Participants mentioned increasing the use of vapes or postponing attempts to stop vaping until they felt less stressed. This was also a recurring finding for participants’ who increased smoking during COVID-19 as it was used to battle boredom during lockdowns [28].

The goal of this study was to understand the determinants and drivers of e-cigarette use and to generate empirical evidence to inform intervention programs and policies aimed at preventing and controlling the use of ENDS products by young adults. By understanding what drives young adults to start using these products, evidence-based policies, regulations, and intervention programs targeting these drivers, can be put into place to tackle the growing use of e-cigarettes.

The findings of this study show the impact of flavoring on vaping initiation. Implementing policies banning flavored tobacco could help tackle this issue. A systematic review of qualitative studies examining the perceptions of and experiences with flavored tobacco products found that flavored products were perceived as less harmful than cigarettes, further supporting the banning of flavored tobacco products, especially as it revolves around curbing smoking initiation among youths [29]. Although there are bans, in the US for example, on flavored cigarettes except for menthol, there are currently no restrictions on flavors in e-cigarettes [30]. Banning flavors has also been advised when considering waterpipe use, which e-cigarette use has frequently been compared to [22].

Banning the advertisement and promotion of e-cigarettes, especially on social media platforms, is also pivotal in decreasing the exposure of these products to youths. Featuring these products in mainstream media and portraying them in a favorable light further strengthens their social acceptability [26]. Furthermore, advertisements also promote the idea that ENDS are healthier alternatives to cigarettes, a belief which may have been adopted by consumers. Advocating for better educational initiatives that raise awareness and highlight the negative health impacts of ENDS is important to prevent smokers from switching or initiating the use of these products. As an alternative, evidence-based cessation programs need to be put into place so that smokers can quit smoking using effective smoking cessation strategies.

Raising cigarette prices has proven to be an effective measure in decreasing their consumption [31]. Increasing sale costs have also been suggested for waterpipe and e-cigarette use regulation [32,33]. The same could be done for all similar products. The careful monitoring of this needs to be considered, as some participants mentioned that, since e-cigarettes were no longer affordable to them, they went back to smoking tobacco cigarettes. Hence, this is something that needs to be taken into consideration when implementing and developing these policies and to avoid substitution between products.

This study is not without its limitations. The first limitation is related to the use of convenience and snowball sampling. Although these methods are cost- and time-efficient, they can result in selection bias [34]. Despite these shortcomings, these sampling methods suited the purpose of this study, which was not to generalize findings, but to enrich the understanding of e-cigarette and HTP use in Lebanon, a country in which little to no research has been done on novel tobacco devices. We note that we set out to understand determinants among young adults regardless of their smoking status, thus one third of the sample provided their perceptions regarding determinants as non-users.

Another possible limitation is the use of Zoom (video conferencing) for data collection. Until recently, conducting interviews online has often been seen as a disadvantage, with the main cited challenge being the establishment of a rapport with the participants [35]. However, in a study investigating the use of Zoom in qualitative research, participants reported feeling more comfortable speaking their mind while in their own safe space [36]. Online interviews were also found to provide greater accessibility to participants [36]. With online interviews, the researcher overcomes the logistical concerns of the participants, and the lack of the need for travel increases the flexibility in the timing and length of interviews [36].

Finally, the possibility of demographic selection bias can be seen as a disadvantage of recruiting through social media, with only younger participants being reached [37]. However, studies have also found that recruitment through social media can eliminate barriers to reaching participants [38], which is pertinent, especially seeing as this study targeted youths.

While this study contributes important data on the determinants of e-cigarette use in Lebanon, more research is needed to investigate the motivations and behavior of e-cigarette users if effective policies and regulations are to be developed and implemented. Moreover, research on e-cigarette and HTP products needs to be conducted separately, since although they share similar determinants and drivers, they are two distinct products that differ in their internal mechanisms—e-cigarettes work by vaporizing e-liquid and HTPs work by heating tobacco. This distinction needs to be made clear during qualitative data collection as some participants may mention both during their discussions when only one or the other is under investigation.

## 5. Conclusions

Both e-cigarettes and IQOS products were seen as merely other modes of smoking, with the use of both products sometimes sharing similar determinants and drivers. Overall, this study was able to identify the most prominent determinants for the initiation of smoking novel tobacco products. The positive perception toward e-cigarettes raises a lot of public health concerns. These key findings have important implications for tobacco monitoring systems, government policies and regulations, as well as public health initiatives in controlling the marketing and advertising of new and emerging tobacco products.

## Figures and Tables

**Table 1:** Characteristics of interview participants.

		Frequency	Percentage
Gender	Female	12	57%
	Male	9	43%
Age	19–21	5	24%
	22–24	3	14%
	25–27	8	38%
	28–30	5	24%
Highest Level of Education	University graduate/Bachelor’s degree	14	67%
	Master’s degree/more	6	28%
	High school graduate	1	5%
Currently Working	Yes	14	67%
	No	7	33%
Smoking Status	E-cigarette users	9	43%
	IQOS users	4	19%
	Non-user of nicotine and tobacco products	8	38%
Total		21	100%

## Data Availability

Data for this study are not publicly available due to the privacy protection concerns of the study participants.
